# The effect of rope jumping training on the dynamic balance ability and hitting stability among adolescent tennis players

**DOI:** 10.1038/s41598-023-31817-z

**Published:** 2023-03-23

**Authors:** Zuozheng Shi, Shu Xuan, Yue Deng, Xinru Zhang, Long Chen, Binglong Xu, Bing Lin

**Affiliations:** 1 School of Physical Education , Chongqing Preschool Education College, Chongqing, China; 2 School of Physical Education, Liaoning Normal University, Dalian, Liaoning China; 3 School of Psychology, Liaoning Normal University, Dalian, Liaoning China

**Keywords:** Health care, Health occupations

## Abstract

To discuss the effects of 12 weeks of jump rope training on dynamic balance and stroke stability in junior tennis players. Sixteen junior tennis players at CTN7 level were used as subjects and randomly divided equally into ExG and ConG for 12 weeks of training, 3 times a week. The ExG performed Jump Rope training and Special Preparation Activity (JR + SPA), the ConG performed only Special Preparation Activity (SPA) to ensure that the training intensity was basically the same. The Biodex Balance System (BBS) was applied to examine the participants' dynamic balance ability, including the Limits of Stability (LOS) and the Single Leg Stability Test (SLST), the International Tennis Number (ITN) tests ball striking stability. Experimental data were statistically analyzed using independent samples *t* test, paired samples *t* test, and repeated measures ANOVA test. We observed significant (P < 0.05) differences in limits of stability (LOS) before and after the test in subjects in the ExG and significant (P < 0.05) differences in ODC, BDC, RDC, FLDC, BLDC, and BRDC metrics in the ExG, and a 2 (group: ExG, ConG) × 2 (time: 0w, 12w) repeated measures ANOVA was performed and found significant (P < 0.05) differences in LDC, RDC, FLDC, BLDC, BRDC, and LOST (S) indexes interacted significantly (P < 0.01) in single-leg stability test (SLST) pre- and post-measures were found in subjects in ExG and in MLSI (L) in ExG (P < 0.05), and 2 (group: ExG, ConG) × 2 (time: 0w, 12w) repeated measures ANOVAs were performed and found that OSI (L), MLSI (L), OSI (R), MLSI (R) interaction on indicators was significant (P < 0.05). The difference in hitting stability between subjects in ExG was significant (P < 0.01) at the pre and post test, while the difference in ConG was significant (P < 0.01) at the pre and post test only on the hitting stability test (depth). 2 (Group: ExG, ConG) × 2 (Time: 0w, 12w) repeated measures ANOVA was performed and found a significant interaction between the two (P < 0.01). We suggest to incorporate Forward double-legged alternating jump rope, Reverse double-legged alternating jump rope, Double-legged alternating cross jump rope, Squatting alternating single-legged jump rope into the regular tennis training course with special techniques, which will help the junior tennis players' dynamic balance ability and hitting stability, and can effectively improve the competitive level of junior tennis players.

## Introduction

Modern tennis competition is characterized by fast pace, high power, strong rotation and multiple tactics, and the frequency of players anticipating, moving, adjusting and being forced to hit the ball has increased, and the balance ability and stability of hitting the ball have received attention from the majority of scholars. Dynamic balance refers to the body's ability to instinctively regulate the body to maintain a certain posture of relative stability when moving or subjected to external forces^1–3^. It is influenced by the height of the center of gravity, the size of the support surface and stability factors^4^, and depends on the coordination of visual, proprioceptive and vestibular information in the central system and the control of motor effectors^5^, and is an important indicator of the specific quality of the tennis player^6^. Some studies have shown that the maintenance of human dynamic balance is mainly based on the ankle joint^3^, and the improvement of human dynamic balance can effectively improve a player's accuracy of footwork, rationality of body control, and joint stability in fast movement^7^, thus helping tennis players to return incoming balls quickly, accurately, and stably, and even score directly. Research shows that skipping rope training can improve the regulation and control ability of neuromuscular system^8^, to a certain extent, can improve the balance control ability of the trunk and lower limbs in human movement^9^, effectively promote the development of lower limb muscle fitness, strength of waist, abdomen and back, balance fitness^10^ also help to improve coordination ability^11,12^. Jump rope training is a common exercise pattern that is less commonly used in tennis. Participants in tennis training believe that the form of jump rope training has a high similarity to modern competitive tennis forms of muscle work, and both are compound movements performed by subjects in a non-stationary state that require a high level of integration of positional, proprioceptive, and vestibular information regulated by the subject's central nervous system, which may affect stroke stability. Therefore, we suggest that there may be some association between jump rope training and dynamic balance and stroke stability in junior tennis players, but this association has not yet been proven. The 12 weeks is consistent with the guidelines recommended by the American University of Sports Medicine (Chapter 2) and the cycle theory of athletic training. 12 weeks is the basic requirement for a microscopic macrocycle of training and constitutes the essential elements of a training macrocycle in which athletes can build on their initial level of competition and contribute to the improvement of individual athletic ability and competitive athletic ability^13^. Therefore, we designed a 12-week training program to study the effect of jump rope training on dynamic balance and stroke stability of young tennis players in order to enrich the training methods of modern tennis competition.

## Methods

### The person in charge

The person in charge of this experiment has been engaged in college tennis training and coaching for 7 years, a national level player and a national level referee with good tennis training and coaching ability, and he participated in the design and supervision of the whole process of the experiment content.

### Subjects

In this experiment, 16 players with Chinese Tennis Technical Grade 7 (CTN7) level were selected as subjects with reference to the "Chinese Tennis Association Tennis Technical Grade Standards and Assessment Methods (Trial)". They were quite stable in hitting medium-speed balls, but they were not able to do well with any ball, and their ability to control the depth, line and power of hitting the ball lacked good control^14^. They came from three different schools, no special training experience in rope skipping, good physical and mental condition, the whole process was fully agreed by the subjects and their parents, and the time and content of the same training program was conducted by the same coach in a centralized training way, effectively avoiding the influence of other factors on the test results. These 16 subjects were randomly and equally divided into an experimental group (ExG, n = 8) and a control group (ConG, n = 8) with the basic profiles shown in Table 1.

**Table 1:** Basic information of subjects.

Group	Age (age)	Height (cm)	Weight (kg)	Body mass index (kg/ index)	Training years (years)
ExG	13.7 ± 0.39	172.8 ± 2.03	63.15 ± 3.43	21.12 ± 0.85	4.31 ± 0.20
ConG	13.6 ± 0.33	173.5 ± 1.12	64.36 ± 2.47	21.41 ± 0.71	4.40 ± 0.25
P value	> 0.05	> 0.05	> 0.05	> 0.05	> 0.05

No significant difference P > 0.05.

### Experimental content

Jumping rope (JR) training content for forward double leg alternating jump rope, reverse double leg alternating jump rope, double leg alternating cross jump rope, squatting alternating single leg alternating jump rope, these experimental elements should be completed while the body remains in motion.

### Forward double-legged alternate jump rope

This experimental content practice time is 120 s, the number of continuous practice is not less than 300 times, practice intensity is about 140–160 r/min, after a gap of 30 s to practice the next content. This action must keep the body upright, from the back of the body through the top of the head to the front of the body to complete the hands, feet together with the bent knee leg lift and empty over the rope practice, in any direction to move.

### Reverse double-legged alternate jump rope

This experimental content practice time is 120 s, the number of exercises is not less than 120 times, practice intensity is about 120–140 r/min, after a gap of 30 s to practice the next content. This action must keep the body upright, from the front of the body through the top of the head to the back of the body to complete the hands, feet together with the bent knee leg lift and empty over the rope practice, in any direction to move.

### Double-legged alternate cross jump rope

This experimental content practice time is 120 s, the number of exercises is not less than 120 times, practice intensity is about 120–140 r/min, after a gap of 30 s to practice the next content. This action must keep the body upright, from the front of the body through the top of the head to the back of the body to complete the hands, feet together with the bent knee leg lift and empty over the rope practice, in any direction to move.

### Squatting alternate single-leg jump rope

This experimental content practice time is 120 s, the number of exercises is not less than 60 times, the intensity of exercise is about 140–160 r/min, after a gap of 30 s to start special training (SPA). This action must keep the body upright, the body squatting to complete a left or right side of the body alternate rope shaking, upright to complete a left leg or right leg alternate rope jumping, moving in any direction to carry out.

Special Preparation Activity (SPA) training content is split leg pad step, side slide step, cross step, broken step, retreat slide step, curve retreat step, backward side slide step, heel lift jump, training intensity is about 120–160 r/min.

### Experimental protocol

In this experiment, the subjects in the ExG group received jump rope training and special preparatory activities (JR + SPA) 3 times a week for 12 weeks, and the jump rope training content was forward double-legged alternating jump rope, backward double-legged alternating jump rope, double-legged alternating cross jump rope, and squatting alternating single-legged jump rope; the ConG received only the regular special preparatory activity (SPA) training content. During the preparation period, the subjects underwent 2 sessions of acclimatization training to facilitate familiarization with the training content and testing procedures, and after an interval of 1 day, all subjects were tested for dynamic balance and ball impact stability (pre-test). The program was continued for 12 weeks, and all subjects were tested for dynamic balance and ball impact stability (post-test) after an interval of 48 h. Because the subjects were trained for only 12 weeks of the microscopic macrocycle, which was relatively short, no interim tests were designed, which could hardly be the main factor influencing the experimental results even though the subjects were in a rapid growth phase, thus ensuring the validity of the experimental results. We paid special attention to the integrity and relevance of the experimental sessions and exercises throughout the training process to avoid youth sports injuries, and there were no cases of subjects dropping out of the experiment due to injury during the 12-week microscopic macrocycle training, and each training session was divided into three parts, the specific contents and schedule are shown in Table 2.

**Table 2:** Experiment content and schedule table.

Group	Part I		Part II		Part III
	Preparation activity (10 min)	Experiment content (20 min)	Single technical (40 min)	Combination technical (40 min)	Stretching and relaxation (10 min)
ExG	Jogging and stretching (10 min)	JR + SPA (20 min)	Hit the straight ball with the forehand	Forehand hit straight ball and backhand hit diagonal ball	Stretching and relaxation exercises
			Hit a straight ball with a backhand		
ConG		SPA (20 min)	Hit the diagonal ball with the forehand	Forehand straight oblique ball and backhand straight ball	
			Hit the diagonal ball with the backhand		

Part I: Subjects in the ExG and ConG performed jogging and stretching exercises together for 10 min, the ExG performed RJ + SPA with 10 min of experimental content and 10 min of SPA content, and the ConG performed SPA training content only for 20 min.

Part II: The same single technique and technique combination exercises. The single technique includes hitting the straight ball with the forehand, hitting the straight ball with the backhand, hitting the diagonal ball with the forehand, hitting the diagonal ball with the backhand. The technique combination exercises include hitting the straight ball with the forehand and hitting the diagonal ball with the backhand, hitting the straight ball with the forehand and hitting the straight ball with the backhand, for 80 min.

Part III: The same stretching and relaxation exercises for 10 min.

### Dynamic balance ability test

The Biodex Balance System (BBS) was used to test the Limits of Stability (LOS) and Single Leg Stability Test (SLST) of the athletes to evaluate the dynamic balance of the subjects. All subjects tested the number of groups, content, time, difficulty and posture to maintain the same, the whole process in accordance with the Biodex Balance System (BBS) operating system instructions, 1 person to explain and supervise, 1 person to record. All subjects completed the limits of stability (LOS) test of body movement and control of random target movement in an unsupported state, and the test was averaged three times, with 10 s for each measurement and 10 s for each interval, including the random target movement of overall direction control (ODC), limits of stability time (LOS-T), forward direction control (FDC), backward direction control (BDC), left direction control (LDC), right direction control (RDC), forward left direction control (FLDC), forward right direction control (FRDC), backward left direction control (BLDC), backward right direction control (BRDC), a total of eight indicators of body control ability test, the higher the value indicates the better control ability. All subjects completed the single leg stability test (SLST) with one leg supported on a non-fixed balance board, controlling the body center of gravity near the center of the cross coordinates in the display for 10 s, with 10 s interval each time, and averaged three times, including the overall stability index (OSI), the anterior–posterior stability index (APSI), and the medial and lateral stability index (MLSI) indicators, the smaller the value indicates better stability. We believe that the pre-test results during the Single Leg Stability Test (SLST) can reflect the existence of dominant and non-dominant foot, and if the stability of one foot on the ipsilateral side of the club hand is significantly higher than that on the opposite side, then the ipsilateral foot of the club hand is considered the dominant foot.

### Hitting stability test

**Figure 1:**
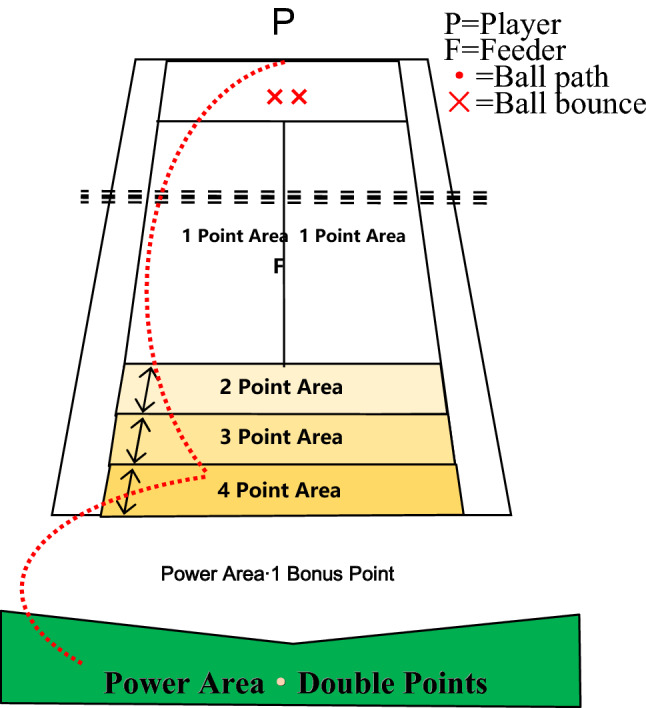
Hitting stability test (depth).

**Figure 2:**
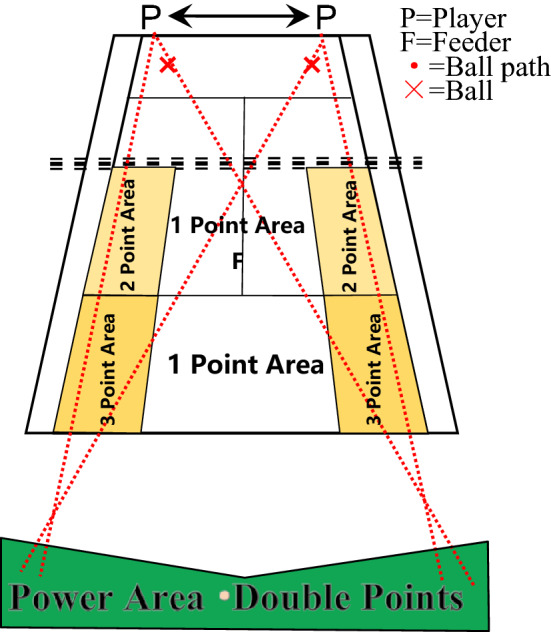
Hitting stability test (accuracy).

The ITN testing system was used for stroke stability, which can comprehensively and objectively evaluate the actual level of the subject with high accuracy and measure the subject's strengths and weaknesses^15^. The ITN testing system was used to test the depth and accuracy of 16 subjects' forehand and backhand baseline strokes, and to improve the effectiveness of the test, the ball drop point was controlled near the singles sideline and baseline, with the aim of mobilizing a wider range of subjects to hit the ball under unstable conditions. Each subject hit 24 balls, including 6 each of the forehand and backhand stroke depth and accuracy tests, with corresponding scores for the ball's first landing point and second landing area, with higher scores indicating better stroke stability. As shown in Figs. 1 and 2.

### Statistical methods

Data were analyzed using SPSS 25. The results of each test were normally distributed and expressed as mean ± standard deviation (M ± SD). The athletes' limits of stability (LOS) and single-leg stability test (SLST) were analyzed by independent samples and paired samples *t* test, and finally 2 × 2 repeated measures ANOVA test was performed to view the effect size. The test results were marked with "*" to indicate significant differences within groups (P < 0.05), "**" to indicate highly significant differences within groups (P < 0.01), and "#" to indicate significant differences between groups (P < 0.05), "##" to indicate highly significant difference between groups (P < 0.01), and no significant difference (P > 0.05).

### Ethical approval

All date used in the study were from published date. This study involved 3 schools. All test procedures and content in this study were approved by the ethical review committee of Chongqing Preschool Education College, all subjects participated voluntarily, all data used were obtained and reviewed in strict accordance with the test protocol, and informed consent was obtained from all subjects, and all data were recorded and used anonymously to avoid disclosure of subjects data.

### Experimental method statement

This study followed the International Tennis Federation approved International Tennis Number test criteria and experimental procedures^15^. The study was conducted according to the experimental methods and requirements of Declaration of Helsinki^16^. And it meets the requirements of Chongqing Preschool Education College for experimentation.

## Results

### Comparative analysis of subjects' limits of stability (LOS) test results

Table 3. To ensure the pre-test levels of LOS in subjects in the ExG and ConG, we conducted an independent samples *t* test and found no significant difference between the ExG and ConG (P > 0.05). After 12 weeks of training, we conducted paired-samples *t* tests on pre- and post-test results for ExG and ConG subjects. It was found that the ExG differed significantly (P < 0.05) in pre-post measurements, while the ConG differed significantly (P < 0.05) only in BDC, LDC, RDC, FRDC, BLDC, and LOST (S) indexes in pre-post measurements. However, we found significant differences (P < 0.05) in ODC, BDC, RDC, FLDC, BLDC, BRDC, and LOST (S) indexes in the ExG. This indicates that jump rope training played a more effective role in improving the limits of stability of the subjects in the ExG.

**Table 3:** Comparative analysis of subjects' limits of stability (LOS) test results.

Test index	ExG			ConG		
	0W	12W	P-value	0W	12W	P-value
ODC (%)	44.35 ± 6.53	58.95 ± 8.63**	< 0.01	44.51 ± 6.74	50.57 ± 8.35	> 0.05
FDC (%)	46.50 ± 4.47	52.12 ± 5.41^#^	> 0.05	43.75 ± 3.83	46.62 ± 3.11	> 0.05
BDC (%)	32.12 ± 3.37	43.25 ± 3.34**	< 0.01	35.50 ± 4.55	43.12 ± 3.98*	< 0.05
LDC (%)	49.50 ± 5.71	67.37 ± 5.87**^,##^	< 0.01	50.13 ± 3.44	58.12 ± 3.17**	< 0.01
RDC (%)	51.37 ± 4.76	67.75 ± 5.14**	< 0.01	55.12 ± 5.59	63.02 ± 4.74*	< 0.05
FLDC (%)	41.62 ± 3.46	58.12 ± 4.59**^,##^	< 0.01	39.38 ± 4.32	43.25 ± 3.49	> 0.05
FRDC (%)	46.87 ± 4.62	63.00 ± 5.80**	< 0.01	49.12 ± 3.78	61.12 ± 5.18**	< 0.01
BLDC (%)	36.50 ± 4.38	51.75 ± 5.69**^,##^	< 0.01	35.51 ± 4.00	40.12 ± 2.02*	< 0.05
BRDC (%)	50.37 ± 4.35	68.25 ± 4.86**^,##^	< 0.01	47.62 ± 3.77	49.25 ± 3.79	> 0.05
LOST (S)	98.63 ± 3.46	87.25 ± 3.07**	< 0.01	94.37 ± 3.73	89.62 ± 2.11**	< 0.01

Within-group differences *P < 0.05, **P < 0.01; Differences between groups ^#^P < 0.05, ^##^P < 0.01.

Table 4. We performed (group: ExG, ConG) × 2 (time: 0w, 12w) repeated measures ANOVA on the subjects' limits of stability (LOS). The results showed that there was a nonsignificant main effect of group for the ODC indicator (F = 1.998, P = 0.179, ES = 0.125), a significant main effect of time (F = 17.559, P = 0.001, ES = 0.556), and a non-significant interaction effect of time and group (F = 2.998, P = 0.105, ES = 0.176). The main effect of FDC indicator group was significant (F = 6.651, P = 0.022, ES = 0.322), the main effect of time was significant (F = 8.821, P = 0.01, ES = 0.387), and the interaction effect of time and group was not significant (F = 0.930, P = 0.351, ES = 0.062). The main effect of BDC indicator group was not significant (F = 3.942, P = 0.067, ES = 0.220), the main effect of time was significant (F = 29.113, P < 0.001, ES = 0.675), and the interaction effect of time and group was not significant (F = 1.021, P = 0.329, ES = 0.068), there was a significant main effect of group (F = 5.917, P = 0.029, ES = 0.297), a significant main effect of time (F = 69.208, P < 0.001, ES = 0.832), and a significant interaction effect of time and group (F = 10.080, P = 0.007, ES = 0.419) for the LDC index. Due to the significant interaction effect, further simple effect analysis was performed and the difference between ExG (49.50%) and ConG (50.13%) at 0W was not significant, and at 12W ExG (67.37%) was significantly higher than ConG (58.12%); ExG and ConG were significantly higher than 0W at 12W. The main effect of RDC indicator group was not significant (F = 0.064, P = 0.804, ES = 0.005). The main effect of time was significant (F = 55.200, P < 0.001, ES = 0.798) and the interaction effect between time and group was significant (F = 6.735, P = 0.021, ES = 0.325). Due to the significant interaction effect, further simple effect analysis was performed and the difference between ExG (51.37%) and ConG (55.12%) was not significant at 0W, and both ExG and ConG were significantly higher than 0W at 12W. The main effect of FLDC indicator group was significant (F = 29.883, P < 0.001, ES = 0.681). The main effect of time was significant (F = 67.167, P < 0.001, ES = 0.828) and the interaction effect between time and group was significant (F = 25.840, P < 0.001, ES = 0.649). Due to the significant interaction effect, further simple effect analysis was performed and the difference between ExG (41.62%) and ConG (39.38%) at 0W was not significant, and at 12W ExG (58.12%) was significantly higher than ConG (43.25%); both ExG and ConG were significantly higher than 0W at 12W. The main effect of FRDC indicator group was not significant (F = 0.012, P = 0.915, ES = 0.001), the main effect of time was significant (F = 64.494, P < 0.001, ES = 0.822), and the interaction effect of time and group was not significant (F = 1.395, P = 0.257, ES = 0.091). The main effect of BLDC indicator group was significant (F = 31.586, P < 0.001, ES = 0.693). The main effect of time was significant (F = 30.626, P < 0.001, ES = 0.686) and the interaction effect between time and group was significant (F = 8.803, P = 0.01, ES = 0.386). Due to the significant interaction effect, further single effect analysis was performed and the difference between ExG (36.50%) and ConG (35.51%) at 0W was not significant and at 12W ExG (51.75%) was significantly higher than ConG (40.12%); ExG was significantly higher than 0W at 12W. The main effect of BRDC indicator group was significant (F = 54.506, P < 0.001, ES = 0.796). The main effect of time was significant (F = 41.750, P < 0.001, ES = 0.749) and the interaction effect between time and group was significant (F = 28.940, P < 0.001, ES = 0.674). Due to the significant interaction effect, further simple effect analysis was performed and the difference between ExG (50.37%) and ConG (47.62%) at 0W was not significant, and at 12W ExG (68.25%) was significantly higher than ConG (49.25%); ExG was significantly higher than 0W at 12W. The main effect of LOST indicator group was not significant (F = 0.063, P = 0.45, ES = 0.041). The main effect of time was significant (F = 65.590, P < 0.001, ES = 0.824) and the interaction effect between time and group was significant (F = 11.099, P = 0.005, ES = 0.442). Due to the significant interaction effect, further simple effect analysis was performed and the difference between ExG (98.63 s) and ConG (94.37 s) at 0W was not significant and both ExG and ConG were significantly higher than 0W at 12W.

**Table 4:** Subjects' limits of stability (LOS) 2 × 2 repeated measures ANOVA test.

Test index	ExG		ConG		Group		Time		Interaction	
	0W	12W	0W	12W	Main effect F-value (ES)	P-value	Main effect F-value (ES)	P-value	Interaction F-value (ES)	P-value
ODC (%)	44.35 ± 6.53	58.95 ± 8.63	44.51 ± 6.74	50.57 ± 8.35	1.998 (0.125)	0.179	17.559 (0.556)	0.001	2.998 (0.176)	0.105
FDC (%)	46.50 ± 4.47	52.12 ± 5.41	43.75 ± 3.83	46.62 ± 3.11	6.651 (0.322)	0.022	8.821 (0.387)	0.01	0.930 (0.062)	0.351
BDC (%)	32.12 ± 3.37	43.25 ± 3.34	35.50 ± 4.55	43.12 ± 3.98	3.942 (0.220)	0.067	29.113 (0.675)	< 0.001	1.021 (0.068)	0.329
LDC (%)	49.50 ± 5.71	67.37 ± 5.87**^,##^	50.13 ± 3.44	58.12 ± 3.17**	5.917 (0.297)	0.029	69.208 (0.832)	< 0.001	10.080 (0.419)	0.007
RDC (%)	51.37 ± 4.76	67.75 ± 5.14**	55.12 ± 5.59	63.02 ± 4.74**	0.064 (0.005)	0.804	55.200 (0.798)	< 0.001	6.735 (0.325)	0.021
FLDC (%)	41.62 ± 3.46	58.12 ± 4.59**^,##^	39.38 ± 4.32	43.25 ± 3.49*	29.883 (0.681)	< 0.001	67.167 (0.828)	< 0.001	25.840 (0.649)	< 0.001
FRDC (%)	46.87 ± 4.62	63.00 ± 5.80	49.12 ± 3.78	61.12 ± 5.18	0.012 (0.001)	0.915	64.494 (0.822)	< 0.001	1.395 (0.091)	0.257
BLDC (%)	36.50 ± 4.38	51.75 ± 5.69**^,##^	35.51 ± 4.00	40.12 ± 2.02	31.586 (0.693)	< 0.001	30.626 (0.686)	< 0.001	8.803 (0.386)	0.01
BRDC (%)	50.37 ± 4.35	68.25 ± 4.86**^,##^	47.62 ± 3.77	49.25 ± 3.79	54.506 (0.796)	< 0.001	41.750 (0.749)	< 0.001	28.940 (0.674)	< 0.001
LOST (S)	98.63 ± 3.46	87.25 ± 3.07**	94.37 ± 3.73	89.62 ± 2.11**	0.603 (0.041)	0.45	65.590 (0.824)	< 0.001	11.099 (0.442)	0.005

Within-group differences *P < 0.05, **P < 0.01; Differences between groups ^#^P < 0.05, ^##^P < 0.01.

### Comparative analysis of subjects' single leg stability test (SLST) results

Table 5. To ensure the pre-test level of SLST in subjects in the ExG and ConG, we conducted an independent samples *t* test and found no significant difference between the ExG and ConG (P > 0.05). After 12 weeks of training, we conducted a paired samples *t* test on the pre-test and post-test results of subjects in the ExG and ConG, and found that the pre-test and post-test in the ExG differed significantly (P < 0.01), and the MLSI (L) index differed significantly (P < 0.05) only in the ExG, indicating that the jump rope training improved single-leg stability (SLST) in subjects in the ExG compared to the ConG played a more effective role.

**Table 5:** Comparative analysis of subjects' single leg stability test (SLST) results.

Test index	ExG			ConG		
	0W	12W	P-value	0W	12W	P-value
OSI (L)	1.67 ± 0.03	0.73 ± 0.10**^,##^	< 0.01	1.76 ± 0.11	1.57 ± 0.08*	< 0.05
APSI (L)	1.73 ± 0.05	1.52 ± 0.08**	< 0.01	1.71 ± 0.09	1.54 ± 0.09**	< 0.01
MLSI (L)	1.34 ± 0.06^#^	0.88 ± 0.12**^,##^	< 0.01	1.16 ± 0.13	1.10 ± 0.08	> 0.05
OSI (R)	1.76 ± 0.04	0.69 ± 0.12**^,##^	< 0.01	1.73 ± 0.11	1.58 ± 0.12**	< 0.01
APSI (R)	1.33 ± 0.06	1.14 ± 0.06**	< 0.01	1.31 ± 0.05	1.18 ± 0.07**	< 0.01
MLSI (R)	1.89 ± 0.04	0.75 ± 0.04**^##^	< 0.01	1.82 ± 0.09	0.92 ± 0.08**	< 0.01

Within-group differences *P < 0.05, **P < 0.01; Differences between groups ^#^P < 0.05, ^##^P < 0.01.

Table 6. We performed a 2 (group: ExG, ConG) × 2 (time: 0w, 12w) repeated measures ANOVA on the subject's single leg stability test (SLST). The results showed that. OSI (L) indicators had significant group main effects (F = 369.088, P < 0.001, ES = 0.963), significant time main effects (F = 270.854, P < 0.001, ES = 0.951), and significant time and group interaction effects (F = 121.847, P < 0.001, ES = 0.897). Due to significant interaction effects, further simple effects analysis was performed, and the difference between ExG (1.67°) and ConG (1.76°) at 0W was not significant, and ExG (0.73°) was significantly higher than ConG. The main effect of APSI (L) indicator group was not significant (F = 0.006, P = 0.938, ES = 0), the main effect of time was significant (F = 40.198, P < 0.001, ES = 0.742), and the interaction effect between time and group was not significant (F = 0.350, P = 0.564, ES = 0.024), the main effect of MLSI (L) indicator group was not significant (F = 0.305, P = 0.589, ES = 0.021), the main effect of time was significant (F = 44.044, P < 0.001, ES = 0.759), and the interaction effect between time and group was significant (F = 25.872, P < 0.001, ES = 0.649). Due to significant interaction effects, further simple effects analysis was performed, and the difference between ExG (1.34°) and ConG (1.16°) at 0W was not significant, and ExG (0.88°) was significantly higher than ConG (1.10°) at 12W; ExG was significantly higher than 0W at 12W. OSI (R) indicators had significant group main effects (F = 91.385, P < 0.001, ES = 0.867), significant time main effects (F = 563.461, P < 0.001, ES = 0.976), and significant time and group interaction effects (F = 321.698, P < 0.001, ES = 0.958). Due to significant interaction effects, further simple effects analysis was performed, and the difference between ExG (1.76°) and ConG (1.73°) at 0W was not significant, and ExG (0.69°) was significantly higher than ConG (1.58°) at 12W; ExG and ConG were significantly higher than 0W at 12W. The APSI (R) index had a non-significant main effect of group (F = 0.392, P = 0.541, ES = 0.027), a significant main effect of time (F = 92.278, P < 0.001, ES = 0.868), and a non-significant interaction effect between time and group (F = 3.145, P = 0.098, ES = 0.183), the main effect of MLSI (R) indicator group was not significant (F = 3.861, P = 0.07, ES = 0.216), the main effect of time was significant (F = 2615.265, P < 0.001, ES = 0.995), and the interaction effect between time and group was significant (F = 35.622, P < 0.001, ES = 0.718). Due to significant interaction effects, further simple effects analysis was performed, and the difference between ExG (1.89°) and ConG (1.82°) at 0W was not significant, and ExG (0.75°) was significantly higher than ConG (0.92°) at 12W; the ExG and ConG were significantly higher than 0W at 12W.

**Table 6:** Subjects' single-leg stability test (SLST) 2 × 2 repeated measures ANOVA test.

Test index	ExG		ConG		Group		Time		Interaction	
	0W	12W	0W	12W	Main effect F-value (ES)	P-value	Main effect F-value (ES)	P-value	Interaction F-value (ES)	P-value
OSI (L)	1.67 ± 0.03^#^	0.73 ± 0.10**^,##^	1.76 ± 0.11	1.57 ± 0.08**	369.088 (0.963)	< 0.001	270.854 (0.951)	< 0.001	121.847 (0.897)	< 0.001
APSI (L)	1.73 ± 0.05	1.52 ± 0.08	1.71 ± 0.09	1.54 ± 0.09	0.006 (0)	0.938	40.198 (0.742)	< 0.001	0.350 (0.024)	0.564
MLSI (L)	1.34 ± 0.06^#^	0.88 ± 0.12**^,##^	1.16 ± 0.13	1.10 ± 0.08	0.305 (0.021)	0.589	44.044 (0.759)	< 0.001	25.872 (0.649)	< 0.001
OSI (R)	1.76 ± 0.04	0.69 ± 0.12**^,##^	1.73 ± 0.11	1.58 ± 0.12**	91.385 (0.867)	< 0.001	563.461 (0.976)	< 0.001	321.698 (0.958)	< 0.001
APSI (R)	1.33 ± 0.06	1.14 ± 0.06	1.31 ± 0.05	1.18 ± 0.07	0.392 (0.027)	0.541	92.278 (0.868)	< 0.001	3.145 (0.183)	0.098
MLSI (R)	1.89 ± 0.04	0.75 ± 0.04**^,##^	1.82 ± 0.09	0.92 ± 0.08**	3.861 (0.216)	0.07	2615.265 (0.995)	< 0.001	35.622 (0.718)	< 0.001

Within-group differences *P < 0.05, **P < 0.01; Differences between groups ^#^P < 0.05, ^##^P < 0.01.

### Comparative analysis of subjects' hitting stability test results

Table 7. To ensure the pre-test results of hitting stability for subjects in the ExG and ConG, we conducted an independent samples *t* test and found no significant difference between the ExG and ConG (P > 0.05). After 12 weeks of training, we conducted a paired samples *t* test on the pre- and post-test scores of subjects in the ExG and ConG and found that the difference between the pre- and post-tests in the ExG was significant (P < 0.01). We also found significant differences (P < 0.01) in the hitting stability test (Depth) and the hitting stability test (Accuracy) between subjects in the ExG, suggesting that the 12W jump rope training had a more comprehensive improvement in hitting stability for subjects in the ExG.

**Table 7:** Comparative analysis of subjects' batting stability test results.

Test project	ExG			ConG		
	0W	12W	P-value	0W	12W	P-value
Hitting stability test (Depth)	40.37 ± 3.03	59.12 ± 3.51**^,##^	P < 0.01	39.62 ± 2.73	45.49 ± 2.59**	P < 0.01
Hitting stability test (Accuracy)	34.75 ± 2.10	48.25 ± 1.98**^,##^	P < 0.01	35.12 ± 2.52	38.47 ± 2.73	P > 0.05

Within-group differences *P < 0.05, **P < 0.01; Differences between groups ^#^P < 0.05, ^##^P < 0.01.

Table 8. We performed a 2 (group: ExG, ConG) × 2 (time: 0w, 12w) repeated measures ANOVA on the subjects' batting stability. The results showed that the batting stability test (depth) index had significant group main effects (F = 68.981, P < 0.001, ES = 0.999), significant time main effects (F = 102.303, P < 0.001, ES = 0.880) and significant time and group interaction effects (F = 28.002, P < 0.001, ES = 0.667). Due to the significant interaction effect, further simple effect analysis was performed and the difference between ExG (40.37) and ConG (39.62) at 0W was not significant and at 12W ExG (59.12) was significantly higher than ConG (45.49); ExG and ConG were significantly higher than 0W at 12W. The hit stability test (accuracy) indicators had significant group main effects (F = 35.500, P < 0.001, ES = 0.717), significant time main effects (F = 93.274, P < 0.001, ES = 0.869), and significant time by group interaction effects (F = 33.848, P < 0.001, ES = 0.707). Due to the significant interaction effect, further simple effect analysis was performed and the difference between ExG (34.75) and ConG (35.12) at 0W was not significant, and at 12W the ExG (48.25) was significantly higher than ConG (38.47); ExG and ConG were significantly higher than 0W at 12W.

**Table 8:** Subjects' batting stability 2 × 2 repeated measures ANOVA test.

Test project	ExG		ConG		Group		Time		Interaction	
	0W	12W	0W	12W	Main effect F-value (ES)	P-value	Main effect F-value (ES)	P-value	Interaction F-value (ES)	P-value
Hitting stability test (Depth)	40.37 ± 3.03	59.12 ± 3.51**^,##^	39.62 ± 2.73	45.49 ± 2.59**	68.981 (0.999)	< 0.001	102.303 (0.880)	< 0.001	28.002 (0.667)	< 0.001
Hitting stability test (Accuracy)	34.75 ± 2.10	48.25 ± 1.98**^,##^	35.12 ± 2.52	38.47 ± 2.73*	35.500 (0.717)	< 0.001	93.274 (0.869)	< 0.001	33.848 (0.707)	< 0.001

Within-group differences *P < 0.05, **P < 0.01; Differences between groups ^#^P < 0.05, ^##^P < 0.01.

## Discussion

### Effect of jump rope training on dynamic balance ability

Human dynamic balance can be achieved by reducing the area of support with the ground^7^, which plays a role in preventing falls in the performance of functional tasks and is crucial in the performance of complex athletic tasks in young athletes^17^. The double foot rotation monoshock jump performs a flexion movement of the hip and knee joints during the jumping stomp, and the alternating support of the left and right foot ensures the dynamic stability of the practitioner during the movement^18^. This fast single swing jump rope path mainly develops the fast super-isometric contraction ability of the ankle joint and the stability of the hip and knee joints^19^. Effective activation of the ankle, knee, and hip joints, which are involved in body positioning and balance regulation, improves the control of the body by the nervous system and muscle proprioceptors during movement^9^, especially in the lower limb and trunk muscles^20^. Better dynamic balance is achieved in junior tennis players as a result of adaptive changes in neuromuscular production. It has been suggested that jump rope training improves endurance and strength of the upper and lower extremities, lumbar and abdominal muscles in subjects^21^ and induces muscle fiber hypertrophy, which explains the significant increase in the limits of stability in several indicators in subjects of ExG and ConG. The subjects in the EnG group differed significantly (P < 0.01) in these directions of OSI (L), MLSI (L), OSI (R), and MLSI (R), indicating that the alternating left and right foot support exercises in jump rope training were more conducive to the development of dynamic balance ability, and this continuous transient acceleration with ankle joint conditioning improved the dynamic balance ability of the subjects. The difference (P < 0.05) in the pre-test results of MLSI (L) of the subjects in the EnG group indicates that junior tennis players have a dominant foot problem, which is due to uneven postural development caused by the long-term use of the ipsilateral leg of the racket-holding hand as a source of strength, a phenomenon consistent with the physical morphological characteristics of tennis players.

### Effect of rope skipping training on stroke stability

Jump rope training exists in a routine unstable state that increases the difficulty of balance adjustment of the hip, knee, and ankle joints, and this unstable state of motion provides better physical preparation for the subject's unstable posture when hitting the ball. During rope skipping, muscle groups in the thigh and foot regions are stimulated to perform stretch-shortening cycle (SSC) contractions^22^, which can improve the stretch-shortening cycle (SSC) movement capacity of the lower limbs^23^, while hip-dominated core muscle groups can better maintain body stability and control^24^. The development of stability and dynamic balance in forehand and backhand strokes may be related to the type of muscle work performed in Jump Rope Training, which involves a centrifugal contraction followed by a centripetal contraction when the muscles perform the "lengthening-shortening cycle", which is somewhat similar to the superlong contraction of the muscles^25^. The power generated by the lower limb movement and the upper limb whipping during the forehand and backhand strokes is mainly based on the centrifugal–centripetal contraction of the upper and lower limb muscles, a form of contraction that can generate more power in a shorter period of time compared to the centripetal contraction. As the forward alternating feet jumping rope, backward alternating feet jumping rope has the characteristics of relaxed movement, fast speed and strong rhythm, it is more conducive to the body to quickly get out of the stationary state, to fully prepare for the rapid movement before the forehand and backhand stroke, and to improve the stroke stability (Accuracy) of the stroke in terms of judgment accuracy and timing of the stroke. And alternate feet cross and squatting alternate single-leg jump rope action is relatively complex, mainly through the hip, knee, ankle and shoulder and elbow joint adjustment to complete the upper and lower limb composite action, depending on the muscle centrifugal–centripetal contraction of rapid conversion, and "stretch-shorten the cycle of movement" can be from the action speed, action amplitude, action strength for the forehand and backhand to create favorable conditions, from the muscle work form to ensure the hitting stability of the ball (Depth). This may be the main reason for the significant difference in stroke stability (P < 0.01) between subjects in the EnG group compared to the ConG group after 12 weeks of skipping rope training.

### The relationship between jump rope training to improve dynamic balance ability and stroke stability

Stroke stability in modern competitive tennis relies heavily on the dynamic balance of the body, and only with smooth support can the upper and lower extremities coordinate their efforts and hit high quality returns^24^, There is a significant correlation between dynamic balance and stroke stability^26–28^. Jumping rope requires continuous and synchronized use of the upper and lower body with a high degree of coordination and rhythm^29^. It requires the body to regain balance and propulsion through coordinated movements of the upper and lower body muscles during its continuous execution^30^. And this dynamic balancing ability helps athletes maintain a more stable center of gravity during intentional activities or specific sports^31^. Jump rope is an important means and method to improve the coordination of the human body through a high degree of integration of the human nervous system and sensory system^32^. It is evident that jump rope training has an improving effect on physical coordination, which may explain the significant differences in the post-test LOS results of ConG subjects in BDC, LDC, RDC, FRDC, BLDC, and LOST (S) indices, but it was not confirmed in this study. Since the double-leg exchange rope skipping training is a compound movement performed under the subject's long-term non-stationary state, this type of training under the state of smaller support surface is more in line with the characteristics of modern tennis competition, where the player's center of gravity changes a lot and posture changes quickly. On the one hand, it enhances the central nervous system's ability to integrate visual, proprioceptive, and vestibular information, and on the other hand, the improved balance adjustment of the hip, knee, and ankle joints improves the stability of the hip-dominant core muscle groups and the coordinated exertion of the upper and lower extremities, providing a stable fulcrum for the circular rotational movement of the shoulder joint. Jump rope exercise of lengthening–shortening the cycle muscle work form increases the mobile hitting power, reduce the time of mobile hitting, ensure the body in the process of high-speed movement of the body stability and hitting accuracy, for hitting the ball to be more adequate preparation. Therefore, we believe that jump rope training has a significant effect on improving the dynamic balance and stroke stability of junior tennis players, and the acquisition of this ability is the result of jump rope training-induced neuromuscular adaptation and muscle hypertrophy, which improves the dynamic balance and stroke stability of junior tennis players in terms of body movement, stroke timing and muscle work forms regulated by the nervous system, while the lack of similar stimuli in pure SPA training, the relatively weak body balance control in mobile stroke movement and the incomplete main factors of stroke stability significance.

## Conclusion

12 weeks of jump rope training can effectively improve the dynamic balance and hitting stability of junior tennis players, and the effect of jump rope training and special preparatory activity training is better than special preparatory activity training alone. We suggest to arrange the corresponding load of Forward double-legged alternating jump rope, Reverse double-legged alternating jump rope, Double-legged alternating cross jump rope, Squatting alternating single-legged jump rope training content between the preparatory activity and technical training of each training session in the junior tennis training cycle curriculum to improve the competitive level of junior tennis.

## Data Availability

The raw data supporting the conclusions of this article are provided by the authors without reservation.
